# Computed tomography based evaluation of the association between sphenoid sinus pneumatization patterns and variations of adjacent bony structures in relation to age and gender

**DOI:** 10.1007/s10143-024-02594-8

**Published:** 2024-07-24

**Authors:** Gülay Açar, Ahmet Safa Gökşan, Demet Aydoğdu

**Affiliations:** 1 Department of Anatomy, Faculty of Medicine, Necmettin Erbakan University, Meram, Konya, 42090 Turkey; 2 Department of Anatomy, Faculty of Medicine, Aksaray University, Merkez, Aksaray, 68100 Turkey; 3 Department of Radiology, Faculty of Medicine, Necmettin Erbakan University, Meram, Konya, 42090 Turkey

**Keywords:** Sphenoid sinus pneumatization, Optic canal, Foramen rotundum, Vidian canal, Carotid canal, Computed tomography

## Abstract

Many studies revealed that the sphenoid sinus pneumatization (SSP) affects the protrusion/dehiscence of adjacent structures including optic canal (OC), foramen rotundum (FR), vidian canal (VC), and carotid canal (CC). Knowledge of this relationship bears vital importance to identify the safest surgical route during transsphenoidal procedures. Therefore, we aimed to determine the individualized prevalence of the protrusion/dehiscence of adjacent structures based on sagittal and coronal SSP (SSSP and CSSP) patterns. Computed tomography images of 300 patients were analysed to identify the SSSP and CSSP types, and the protrusion/dehiscence of adjacent structures was determined. The relationship between the variables was examined using statistical analysis in terms of age, gender, and laterality. The most prevalent SSSP type was postsellar (62.7%), followed by sellar (30%), presellar (6.6%), and conchal (0.7%). In 71.3% of patients, five types of CSSP were observed, with 23.6% and 21.7% exhibiting Type IV and V, respectively. Our results indicated that postsellar type, Type IV and V CSSP associated with the highest likelihood of protrusion/dehiscence of OC, FR, VC, and CC. Furthermore, no significant correlation was observed between these qualitative variables and gender, with the exception of the VC dehiscence, the protrusion of OC and CC. No notable differences were identified with respect to laterality. Also, the probability of having postsellar type, Type IV and V CSSP, as well as the protrusion of OC, VC, and CC, decreased with increasing age. Further detailed analysis of this association is required to predict the size of the surgical window and to prevent neurovascular injury.

## Introduction

The sphenoid sinus (SS) is characterized by complex variable cavity locating in the skull base. Compared to other paranasal sinuses, it is the most inaccessible air filled cavity, which reaches radiologically visible adult dimensions at 14 years of age [1, 2]. The degree of sphenoid sinus pneumatization (SSP) ranging from absent to extensive, brings along important clinical and surgical considerations due to its association with numerous morphological variability in the anatomic configuration of vital adjacent neurovascular structures as well as the extent of pneumatization into other portions of the sphenoid bone. The degree of sphenoid sinus pneumatization (SSP) ranges from absent to extensive. This has significant clinical and surgical implications due to its association with a wide range of morphological variations in the anatomic configuration of vital adjacent neurovascular structures, as well as the extent of pneumatization into other portions of the sphenoid bone. Functional endoscopic sinus surgery (FESS) and transsphenoidal procedures allows the surgeon with a technique that provides surgical corridor to access the middle cranial fossa and neighbouring sellar regions through the SS, while preserving adjacent bony structures, such as the optic canal (OC), foramen rotundum (FR), vidian canal (VC), and carotid canal (CC), and resulting in less iatrogenic injury [3–5]. Regardless of the classifications according to sagittal (anteroposterior) and coronal (lateral) planes, detailed understanding of the SSP patterns remains partly subjective. Depending on the position of the SS on sagittal and coronal planes, the SSP patterns can be described as separate two groups; sagittal SSP (SSSP) and coronal SSP (CSSP). In previous studies, SSSP types are described as conchal, presellar, sellar, and postsellar in relation to the anterior and posterior limits of sella turcica (ST), while CSSP types are specified depending on the extent of pneumatization into the greater wing of the sphenoid (GWS), pterygoid process (PP), and anterior clinoid process (ACP) [1–11].

Based on analysis of the relevant literature, it was noted that the higher degree of SSP affects the protrusion and dehiscence of OC, FR, VC, and CC such that it results in a higher dehiscence and protrusion of them into the SS. Numerous authors reported that the pneumatization pattern of the SS and the anatomical variations of vital neurovascular structures are commonly altered by ethnic variability A review of the relevant literature revealed that a higher degree of SSP affects the protrusion and dehiscence of the OC, FR, VC, and CC, resulting in a higher dehiscence and protrusion of these structures into the SS. Numerous authors have reported that the pneumatization pattern of the SS and the anatomical variations of vital neurovascular structures are commonly influenced by ethnic variability. [1, 3, 12–16]. Moreover, there is still a lack of consensus regarding the influence of age and gender on the morphological variability across different SSP types and the association with the protrusion and dehiscence of adjacent structures [2, 17].

With the substantial integration of computed tomography (CT) into surgical interventions, the SS and neighbouring neurovascular structures can be easily viewed and an anatomical variant of these bony structures have been evaluated along with their association with SSP patterns. Precise radioanatomical knowledge of the SSP patterns in relation to the degree of protrusion and dehiscence of surgically relevant vital structures, is of considerable interest to the clinicians particularly those from otorhinolaryngology, radiology and neurosurgery. A detailed analysis of this relationship is needed to predict the size of the surgical window and to choose the safest surgical approach, while preventing iatrogenic injury [1, 3, 18, 19]. In this current study we aimed to determine the individualized prevalence of the protrusion and dehiscence of OC, FR, VC, and CC based on SSSP and CSSP types so as to first, analyze the possible association with adjacent neurovascular structures, and second, elucidate the impact of gender, laterality, and age on these variations.

## Materials and methods

### Study design and inclusion criteria for patients

This retrospective study was approved by the University’s Ethics and Research Committees, with the registration number 2023/4634. Using G power (version 3.1.9.2) with an alpha level of 0.05 and a power of 80%, a minimum sample of 130 subjects was considered sufficient. All paranasal sinus CT scans performed between November 2023 and March 2024 in the database of the Radiology Department of the University were reviewed and images of 300 patients (150 females, 150 males) that met the inclusion criteria were evaluated. The inclusion criteria were age ≥ 18 years, no history of previous trauma or sinus surgery, no having marked maxillofacial deformity or artefact, and images with the SS completely visible. The mean age of patients was 40.78 ± 14.44 with age range of 18–82 years. All images were interpreted independently by an experienced radiologist and anatomist. Inter-rater consistency quantified with the most appropriate statistical test.

### CT imaging and analysis

All images were acquired using Somatom Drive (Siemens Healthineers, Germany) 256 multislice CT with the following study parameters; exposure 120 kV, 74 mA, 60 mAs; rotation time 0.28 s; slice thickness 0.625 mm. All images were examined using a workstation (Snygo Via, Siemens, Germany). Classifications of pneumatization and detection of the OC, FR, VC, and CC variations in each individual sinus in subject half-sides (600 hemi-sinuses) were done.

### Morphological characteristics of the sphenoid sinus pneumatization

**Fig. 1:**
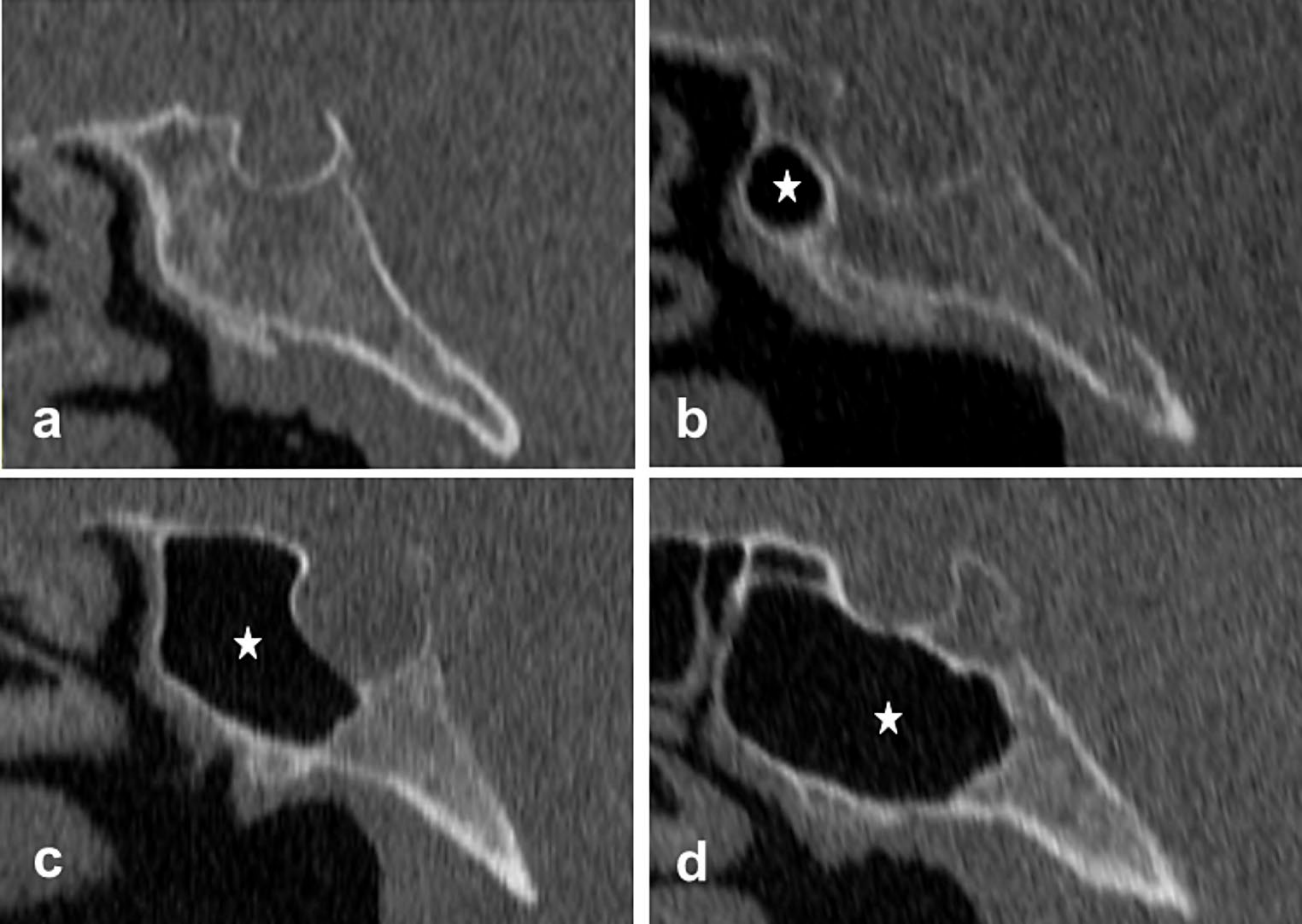
Sagittal CT images show types of sagittal sphenoid sinus pneumatization, **a** conchal, **b** presellar, **c** sellar, **d** postsellar

For the present study, the SSP patterns were classified as two main types based on the observation in coronal and sagittal planes with the support of literature [6–10]. SSSP was divided into four groups in relation to the anterior and posterior walls of the ST [6], namely as follows:

- Conchal type: An absent or small extension of the SSP in front of the anterior wall of the ST (Fig. 1a).
- Presellar type: Posterior limit of the SSP is in front of the anterior wall of the ST (Fig. 1b).
- Sellar type: Posterior limit of the SSP is in front of the posterior walls of ST (Fig. 1c).
- Postsellar type: Posterior limit of the SSP is behind the posterior wall of ST (Fig. 1d).

**Fig. 2:**
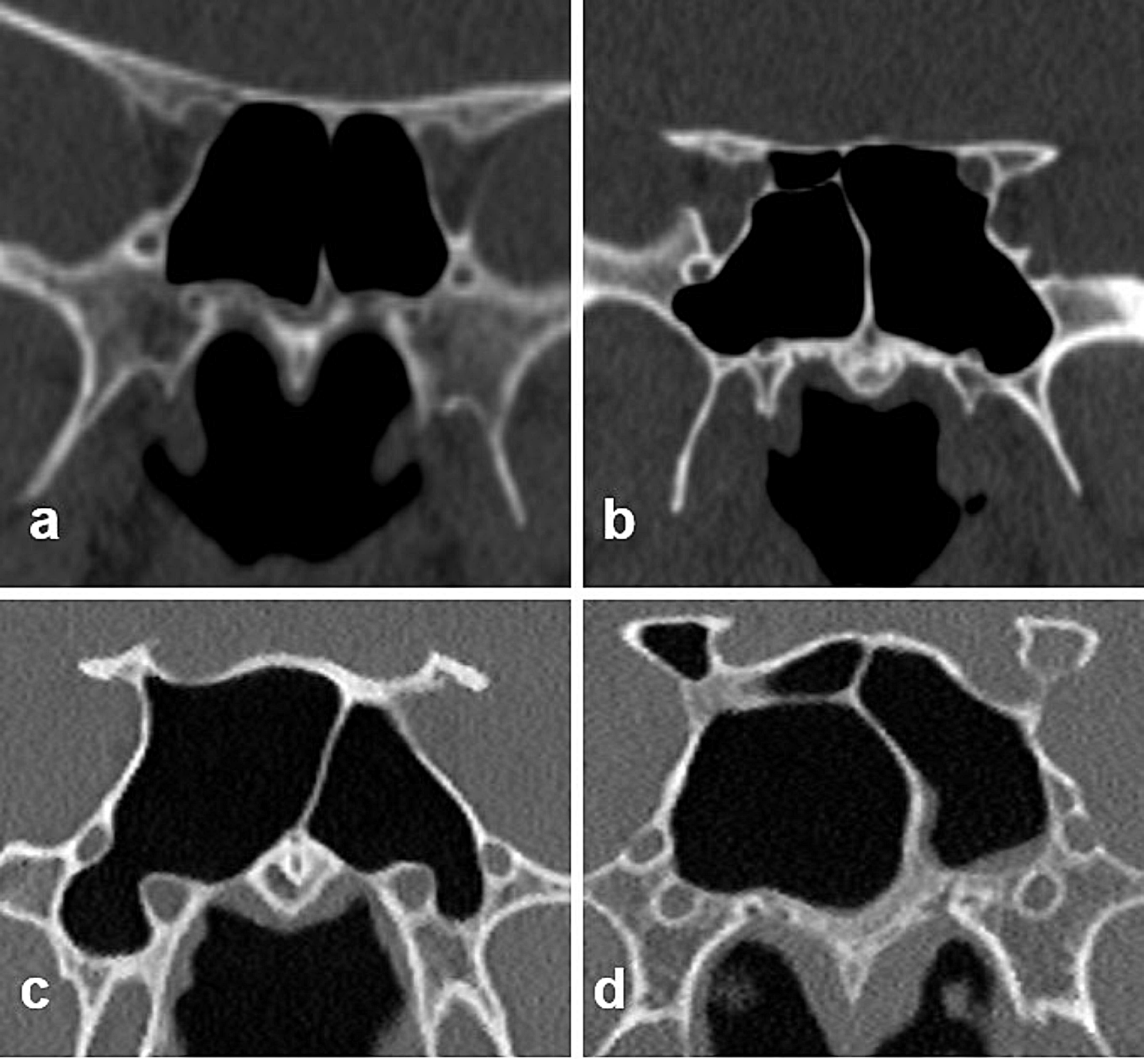
Coronal CT images show types of coronal sphenoid sinus pneumatization, **a** Type 0, **b** Type I, **c** Type II, **d** Type III.

**Fig. 3:**
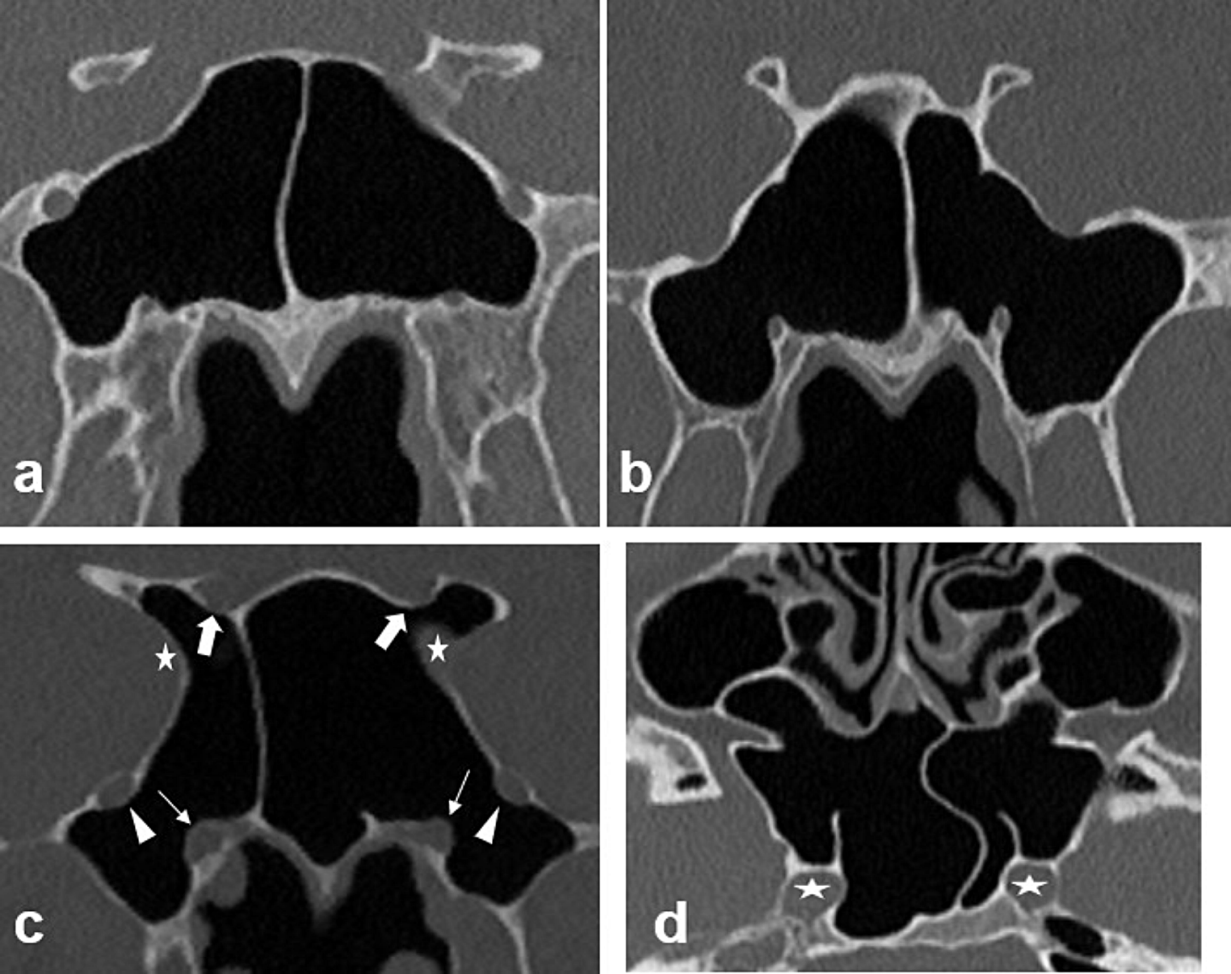
On coronal CT images, **a** Type IV on right side and Type I on left side, **b** bilateral Type IV, **c** bilateral Type V coronal sphenoid sinus pneumatization with bilateral protrusion and dehiscence of vidian canal (white thin arrow), foramen rotundum (white arrowhead), optic canal (white thick arrow), and carotid canal (white star) into the sphenoid sinus. **d** Axial CT image shows bilateral protrusion of carotid canal and the dehiscence of carotid canal on right side (white star)

For the classification of CSSP, three lines (horizontal ACP line, horizontal VC line, and vertical FR line) were identified as the demarcation between the SS, ACP, GWS, and PP [1, 8]. CSSP were classifed into six types in relation to the extent of SSP into the GWS, PP, and ACP:

- Type 0: There are no an aberrant pneumatization in the ACP, PP, and GWS (Fig. 2a).
- Type I (only GWS pneumatization): SSP extends only into the GWS medially or laterally to a vertical line passing the FR (Fig. 2b).
- Type II (only PP pneumatization): SSP extends only into the PP below the horizontal plane passing the VC (Fig. 2c).
- Type III (only ACP pneumatization): SSP extends only into the ACP (Fig. 2d).
- Type IV (combined GWS and PP pneumatization): SSP extends into both the PP and GWS (Fig. 3a).

Type V (combined GWS, PP, and ACP pneumatization): SSP extends into the ACP, PP, and GWS (Fig. 3b).

### Morphological characteristics of adjacent bony structures

The dehiscence (the absence of bone density overlying the canal) and protrusion of the OC, FR, VC, and CC into the SS were classified into two types as absent (No) and present (Yes) (Fig. 3c, d). The prevalence of the SSSP and CSSP patterns and the associated protrusion or dehiscence of the adjacent bony structures stratified by age, gender and laterality were determined by using coronal, sagittal and axial CT images.

### Statistical analysis

Statistical analysis of the study data was performed using SPSS (version 25.0, IBM SPSS Inc., Chicago, IL, USA). Categorical variables and frequency distribution with respect to gender, age groups, and lateralization were given as counts and percentages (%). Cohen’s kappa (*k*) coefficient of agreement with a 95% confidence interval (CI) was determined for inter-observer reliability. The Pearson’s chi-square test, one-way ANOVA (One-way analysis of variance), and logistic regression analysis were used to explore the association between morphological characteristics of SSSP, CSSP, anatomic variants of adjacent structures (absent versus unilateral or bilateral protrusion/dehiscence), and potential influencing factors, such as age, gender, and laterality. Spearman’s correlation coefficient was used to evaluate the correlation between categorical variables. The data were considered statistically significant with *p* < 0.05.

## Results

### Prevalence of the sagittal sphenoid sinus pneumatization types

One female and male patients in this study exhibited conchal type pneumatization (2/300, 0.7%). The most observed was the postsellar type (85 female and 103 male, 188/300, 62.7%), followed by the sellar type (54 female and 36 male, 90/300, 30%) and the presellar type (10 female and 10 male, 20/300, 6.6%). In females; sellar type (36%), and in males; postsellar type (68.6%) were detected more, but there was no significant difference with respect to gender in terms of the incidence of SSSP types (*p* = 0.150, χ2 = 5.323) (Table 1).

**Table 1:** The relationship between the types of sagittal and coronal sphenoid sinus pneumatization, the protrusion, and dehiscence of adjacent structures in females and males

		Female (*n* = 150)	Male (*n* = 150)	*p* value
Sagittal SSP	Conchal	1 (0.7%)	1 (0.7%)	*p* = 0.150
	Presellar	10 (6.7%)	10 (6.7%)	
	Sellar	54 (36%)	36 (24%)	χ^2^ = 5.323
	Postsellar	85 (56.6%)	103 (68.6%)	
Right coronal SSP	Type 0	84 (56%)	83 (55.3%)	***p*** = 0.009* **χ**^**2**^ **= 17.016**
	Type I	10 (6.7%)	24 (16%)	
	Type II	1 (0.7%)	1 (0.7%)	
	Type III	7 (4.6%)	4 (2.6%)	
	Type IV	31 (20.7%)	10 (6.7%)	
	Type V	17 (11.3%)	28 (18.7%)	
Left coronal SSP	Type 0	80 (53.3%)	78 (52%)	*p* = 0.180 χ^2^ = 8.886
	Type I	14 (9.3%)	12 (8%)	
	Type II	0 (0%)	1 (0.7%)	
	Type III	4 (2.7%)	6 (4%)	
	Type IV	29 (19.4%)	23 (15.3%)	
	Type V	23 (15.3%)	30 (20%)	
OC protrusion	Absent	95 (63.4%)	65 (43.4%)	***p*** = 0.013* **χ**^**2**^ **= 6.178**
	Unilateral	17 (11.3%)	26 (17.3%)	
	Bilateral	38 (25.3%)	59 (39.3%)	
OC dehiscence	Absent	83 (55.3%)	80 (53.3%)	*p* = 0.376 χ^2^ = 0.783
	Unilateral	36 (24%)	41 (27.4%)	
	Bilateral	31 (20.7%)	29 (19.3%)	
FR protrusion	Absent	88 (58.7%)	80 (53.3%)	*p* = 0.363 χ^2^ = 0.829
	Unilateral	49 (32.7%)	58 (38.7%)	
	Bilateral	13 (8.6%)	12 (8%)	
FR dehiscence	Absent	111 (74%)	120 (80%)	*p* = 0.477 χ^2^ = 0.505
	Unilateral	29 (19.3%)	22 (14.7%)	
	Bilateral	10 (6.7%)	8 (5.3%)	
VC protrusion	Absent	58 (38.7%)	69 (46%)	*p* = 0.204 χ^2^ = 1.615
	Unilateral	34 (22.6%)	31 (20.7%)	
	Bilateral	58 (38.7%)	50 (33.3%)	
VC dehiscence	Absent	69 (46%)	92 (61.3%)	***p*** = 0.041* **χ**^**2**^ **= 4.164**
	Unilateral	36 (24%)	26 (17.3%)	
	Bilateral	45 (30%)	32 (21.4%)	
CC protrusion	Absent	88 (58.7%)	62 (41.3%)	***p*** = 0.005* **χ**^**2**^ **= 7.792**
	Unilateral	16 (10.6%)	21 (14%)	
	Bilateral	46 (30.7%)	67 (44.7%)	
CC dehiscence	Absent	117 (78%)	124 (82.7%)	*p* = 0.401 χ^2^ = 0.706
	Unilateral	22 (14.7%)	18 (12%)	
	Bilateral	11 (7.3%)	8 (5.3%)	

*Statistically significant p value, χ2 Pearson chi-square test result, *SSP* sphenoid sinus pneumatization types, *OC* optic canal, *FR* foramen rotundum, *VC* vidian canal, *CC* carotid canal

### Prevalence of the coronal sphenoid sinus pneumatization types

Regarding the CSSP types, 28.7% of subjects had a normal pneumatization. In relation to the extent of SSP into the GWS, PP, and ACP, the most common types were Type IV and V (23.6% and 21.7%), whereas the least common was Type II (1%) (Table 2). In females; Type IV (20.7%) and in males; Type V pneumatization (18.7%) were detected more on the right. The prevalences differed significantly between females and males (*p* = 0.009, χ2 = 17.016) (Table 1). Similarly, Type IV and V pneumatization were detected in 19.4% of females and 20% of males on the left side, but no significant difference was observed between the genders (*p* = 0.180, χ2 = 8.886) (Table 1). The prevalence of extensive pneumatization was found to be more pronounced on the left side in both Type IV and Type V. Additionally, Type V was observed to be bilaterally distributed with the greatest frequency (*p* < 0.001). Details about the prevalences of CSSP types were seen in Table 2.

**Table 2:** Distribution of the coronal sphenoid sinus pneumatization types, the protrusion, and dehiscence of adjacent structures according to the laterality

		Right		Left		Bilateral		Total
		n	(%)	n	(%)	n	(%)	n (%)
CSSP	Type I	32	(55.2%)	24	(41.4%)	2	(3.4%)	58 (19.3%)
	Type II	2	(66.7%)	1	(33.3%)	0	(0%)	3 (1%)
	Type III	7	(41.2%)	6	(35.3%)	4	(23.5%)	17 (5.7%)
	Type IV	19	(26.8%)	30	(42.2%)	22	(31%)	71 (23.6%)
	Type V	12	(18.4%)	20	(30.8%)	33	(50.8%)	65 (21.7%)
Protrusion	OC	23	(16.4%)	20	(14.3%)	97	(69.3%)	140 (46.7%)
	FR	51	(38.7%)	56	(42.4%)	25	(18.9%)	132 (44%)
	VC	28	(16.2%)	37	(21.4%)	108	(62.4%)	173 (57.7%)
	CC	18	(12%)	19	(12.7%)	113	(75.3%)	150 (50%)
Dehiscence	OC	48	(35%)	29	(21.2%)	60	(43.8%)	137 (45.6%)
	FR	33	(47.8%)	18	(26.1%)	18	(26.1%)	69 (23%)
	VC	30	(21.6%)	32	(23%)	77	(55.4%)	139 (46.3%)
	CC	18	(30.5%)	22	(37.3%)	19	(32.2%)	59 (19.7%)

*CSSP* coronal sphenoid sinus pneumatization types, *OC* optic canal, *FR* foramen rotundum, *VC* vidian canal, *CC* carotid canal

### Prevalence of protrusion and dehiscence of the adjacent bony structures

The protrusion of the OC, FR, VC, and CC were observed in 46.7%, 44%, 57.7%, and 50% of subjects, whereas the prevalences of the OC, FR, VC, and CC dehiscence were 45.6%, 23%, 46.3%, and 19.7%, respectively. The rate of protrusion did not exhibit a significant change according to the side of the body on which it was observed, whereas the protrusion of the OC, VC, and CC were found to be bilateral in nature (*p* < 0.005). Conversely, the OC and FR dehiscence were observed with greater frequency on the right side, and the dehiscence of the OC and VC was most frequently bilateral (*p* < 0.005) (Table 2). The detailed information regarding the frequency distributions of right, left and bilateral sides for the protrusion and dehiscence of these structures was given in Table 2.

There was a significant difference in the incidence of protrusion of the OC (*p* = 0.013, χ2 = 6.178) and CC (*p* = 0.005, χ2 = 7.792), and the dehiscence of the VC (*p* = 0.041, χ2 = 4.164) according to gender (Table 1). The protrusion of the OC was detected in 85 (56.6%) of the males and 55 (36.6%) of the females. Similarly, the protrusion of the CC was detected in 88 (58.7%) of the males and 62 (41.3%) of the females. In general, unilateral and bilateral dehiscence were more frequently observed in females than in males. However, the prevalence of the VC dehiscence, with 54% (81/150) in females and 38.7% (58/150) in males, demonstrated a significant difference (Table 1).

### Relationships of the SSSP types with CSSP types and the protrusion and dehiscence of the adjacent structures

**Fig. 4:**
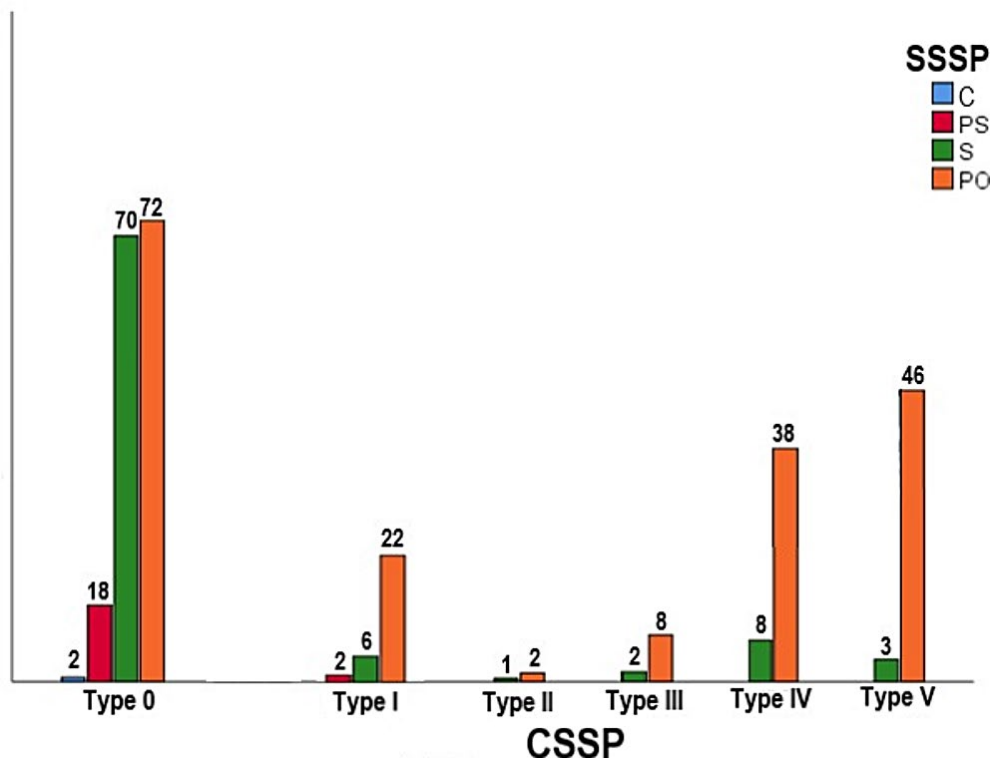
Bar diagram shows the relationship between the types of coronal (CSSP) and sagittal sphenoid sinus pneumatization (SSSP), C conchal (blue), PS presellar (red), S sellar (green), PO postsellar types (orange)

**Fig. 5:**
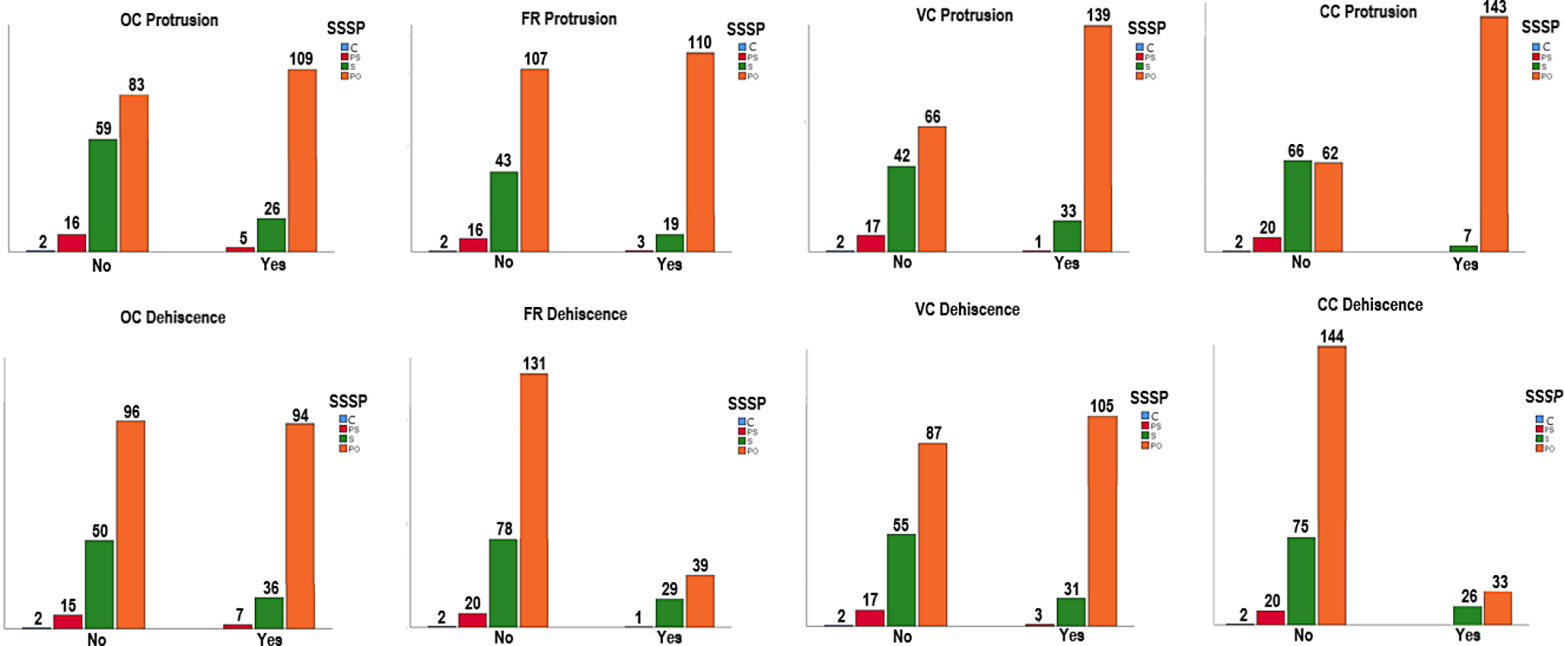
Bar diagram shows the relationship between the protrusion and dehiscence of adjacent structures and the types of sagittal sphenoid sinus pneumatization (SSSP), C conchal (blue), PS presellar (red), S sellar (green), PO postsellar types (orange), OC optic canal, FR foramen rotundum, VC vidian canal, CC carotid canal

We found that the SSSP types had a major impact on the existence of GWS, PP, and ACP pneumatization. The patients with the postsellar type exhibited the highest prevalence of Type I, IV, and V CSSP in comparison to the sellar type (Fig. 4). There was a strong association between the SSSP and CSSP types (*p* = 0.000) (Table 3). The protrusion and dehiscence of adjacent structures depends on the SSSP types (especially postsellar type) with a significant relationship (*p* < 0.005) except the dehiscence of the FR and CC (*p* = 0.476 and *p* = 0.493) (Table 3). The detailed information regarding the frequency distributions of absent, unilateral, and bilateral sides for the protrusion and dehiscence of adjacent structures in SSSP types was given in Table 4. The OC protrusion was observed in 140 (46.6%) of the SSSP types, with no subjects exhibiting this feature in the conchal type, 5 (1.7%) subjects in the presellar type, 26 (8.6%) subjects in the sellar type, and 109 (36.3%) subjects in the postsellar type. Similar results were observed for the other structures as seen in Fig. 5 and a statistical association between these categorical variations can be seen in Table 3. Our results revealed that the prevalence of the protrusions of all structures exhibited a marked increase in postsellar type, whereas the dehiscence of the FR and CC did not demonstrate a significant correlation with the increasing degree of the SSSP. So, an increased pneumatization of the SS has a greater effect on the protrusion rates of surrounding structures than dehiscence (Tables 3 and 4).

**Table 3:** The relationship of sagittal sphenoid sinus pneumatization with the coronal sphenoid sinus pneumatization, the protrusion and dehiscence of adjacent structures

Relationship	*p*	df	CC
OC protrusion and SSSP types	**0.001** *	3	0.349
OC dehiscence and SSSP types	**0.005** *	3	0.248
FR protrusion and SSSP types	**0.005** *	3	0.267
FR dehiscence and SSSP types	0.476	3	0.177
VC protrusion and SSSP types	**0.000** *	3	0.433
VC dehiscence and SSSP types	**0.001** *	3	0.323
CC protrusion and SSSP types	**0.000** *	3	0.590
CC dehiscence and SSSP types	0.493	3	0.156
CSSP types and SSSP types	**0.000** *	5	0.466

* p value shows statistically significant Chi-square test result, *df* degree of freedom, *CC* contingency coefficient degree of association *SSP* sphenoid sinus pneumatization, *OC* optic canal, *FR* foramen rotundum, *VC* vidian canal, *CC* carotid canal

**Table 4:** Distribution of the protrusion and dehiscence of adjacent structures according to sagittal sphenoid sinus pneumatization types

		Presellar		Sellar				Postsellar			Total
		n	(%)	n			(%)	n		(%)	*n* + 2 (conchal)
OC protrusion	Absent	16	(10%)		59	(36.9%)			83	(51.9%)	160 (53.3%)
	Unilateral	2	(4.6%)		11	(25.6%)			30	(69.8%)	43 (14.3%)
	Bilateral	3	(3.1%)		15	(15.5%)			79	(81.4%)	97 (32.3%)
OC dehiscence	Absent	15	(9.2%)		50	(30.7%)			96	(58.9%)	163 (54.3%)
	Unilateral	5	(6.5%)		25	(32.5%)			47	(61%)	77 (25.7%)
	Bilateral	2	(3.3%)		11	(18.3%)			47	(78.4%)	60 (20%)
FR protrusion	Absent	16	(9.5%)		43	(25.6%)			107	(63.7%)	168 (56%)
	Unilateral	3	(2.8%)		6	(5.6%)			98	(91.6%)	107 (35.7%)
	Bilateral	0	(0%)		13	(52%)			12	(48%)	25 (8.3%)
FR dehiscence	Absent	20	(8.6%)		78	(33.7%)			131	(56.8%)	231 (77%)
	Unilateral	1	(2%)		21	(41.2%)			29	(56.8%)	51 (17%)
	Bilateral	0	(0%)		8	(44.4%)			10	(55.6%)	18 (6%)
VC protrusion	Absent	17	(13.4%)		42	(33.1%)			66	(52%)	127 (42.3%)
	Unilateral	0	(0%)		18	(27.7%)			47	(72.3%)	65 (21.7%)
	Bilateral	1	(0.9%)		15	(13.9%)			92	(85.2%)	108 (36%)
VC dehiscence	Absent	17	(10.6%)		55	(34.2%)			87	(54%)	161 (53.7%)
	Unilateral	2	(3.2%)		20	(32.3%)			40	(64.5%)	62 (20.6%)
	Bilateral	1	(1.3%)		11	(14.3%)			65	(84.4%)	77 (25.7%)
CC protrusion	Absent	20	(13.3%)		66	(44%)			62	(41.4%)	150 (50%)
	Unilateral	0	(0%)		3	(8.1%)			34	(91.9%)	37 (12.3%)
	Bilateral	0	(0%)		4	(3.5%)			109	(96.5%)	113 (37.7%)
CC dehiscence	Absent	20	(8.3%)		75	(31.1%)			144	(59.8%)	241 (80.4%)
	Unilateral	0	(0%)		18	(45%)			22	(55%)	40 (13.3%)
	Bilateral	0	(0%)		8	(42.1%)			11	(57.9%)	19 (6.3%)

*OC* optic canal, *FR* foramen rotundum, *VC* vidian canal, *CC* carotid canal

### Relationships of the CSSP types with the protrusion and dehiscence of the adjacent structures

**Fig. 6:**
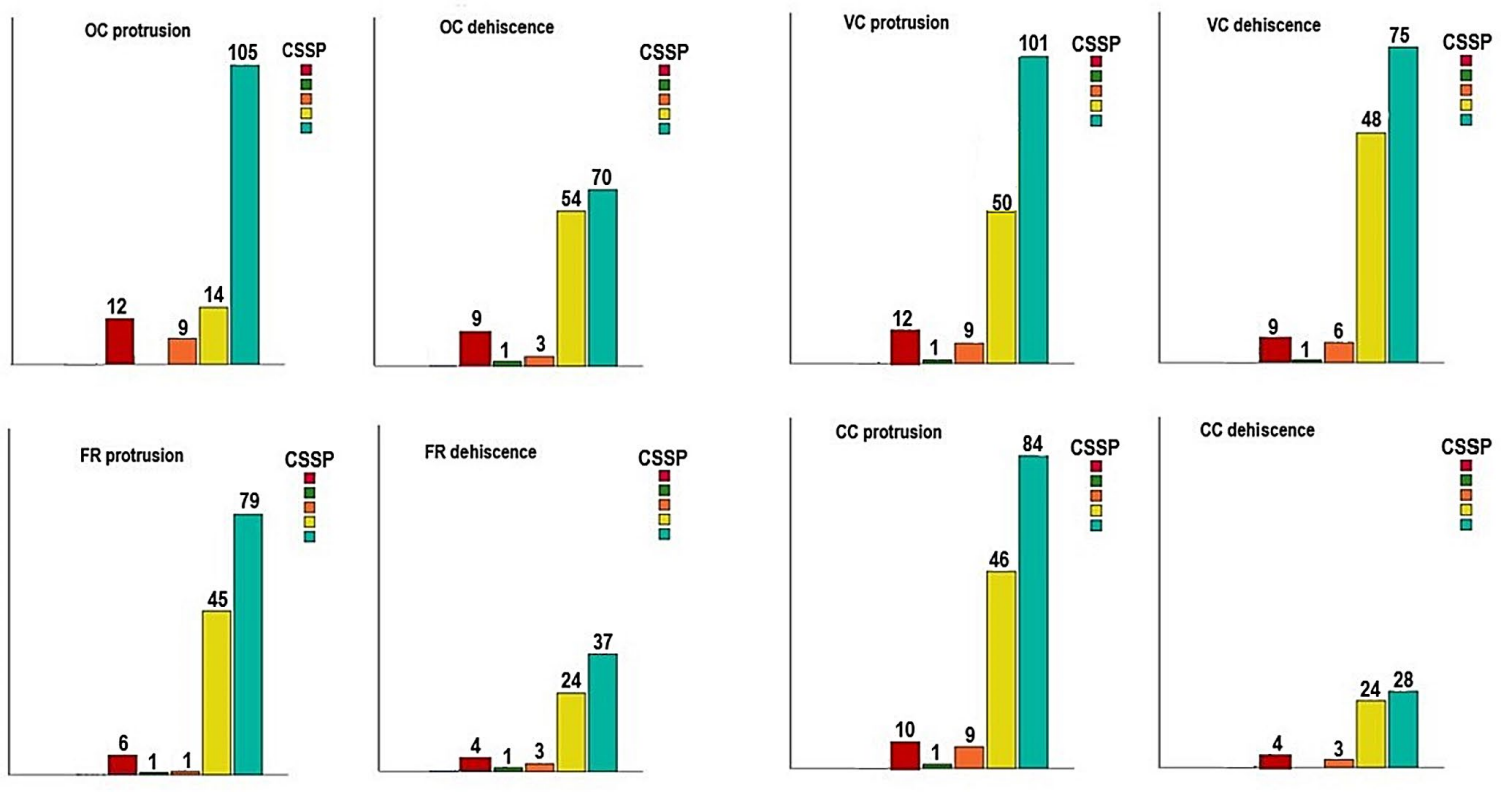
Bar diagram shows the relationship between the protrusion and dehiscence of adjacent structures and the coronal sphenoid sinus pneumatization (CSSP) types, Type I (red), Type II (green), Type III (orange), Type IV (yellow), Type V (blue), OC optic canal, FR foramen rotundum, VC vidian canal, CC carotid canal

The OC protrusion was observed in 140 (46.7%) of the CSSP types, with no subjects exhibiting this feature in Type II, 12 (4%) in Type I, 9 (3%) in Type III, 14 (4.7%) in Type IV, and 105 (35%) in Type V (Fig. 6). The detailed information regarding the number of the protrusion and dehiscence of other adjacent structures in CSSP types was given in Fig. 6 and the statistical association between them can be seen in Table 5. Our findings indicated that the prevalence of the protrusion of the OC is significantly elevated, particularly in Type V. Conversely, the number of protrusions and dehiscences of other structures is markedly increased in Types IV and V. Furthermore, bilateral OC protrusion was observed in 97 (69.3%) patients, with 81 (83.5%) exhibiting Type V, 10 (10.3%) exhibiting Type IV, and 6 (6.2%) exhibiting Type III, respectively. Similarly, among the 108 patients with bilateral VC protrusion, 72 (66.7%) exhibited Type V, 33 (30.5%) exhibited Type IV, and three (2.8%) exhibited other types of CSSP, respectively. Similar results were observed for the CC protrusion. On the other hand, the frequency of bilateral FR protrusion and bilateral dehiscence of all adjacent structures were found equally in Type IV and V. As a consequence of the considerable increase in the protrusions of all structures observed in Types IV and V, we verified that the presence of combined GWS, PP, and ACP pneumatization was associated with a higher frequency of protrusion and dehiscence of adjacent structures (*p* < 0.05), with the exception of FR and CC dehiscence (*p* = 0.482 and *p* = 0.509) (Table 5).

**Table 5:** The relationship of coronal sphenoid sinus pneumatization types with the protrusion and dehiscence of adjacent structures

Relationship	*p*	df	CC
OC protrusion and coronal SSP	**0.000** *	5	0.580
OC dehiscence and coronal SSP	**0.009** *	5	0.212
FR protrusion and coronal SSP	**0.005** *	5	0.244
FR dehiscence and coronal SSP	0.482	5	0.113
VC protrusion and coronal SSP	**0.000** *	5	0.477
VC dehiscence and coronal SSP	**0.002** *	5	0.273
CC protrusion and coronal SSP	**0.001** *	5	0.309
CC dehiscence and coronal SSP	0.509	5	0.115

* p value shows statistically significant Chi-square test result, *df* degree of freedom, *CC* contingency coefficient degree of association *SSP* sphenoid sinus pneumatization, *OC* optic canal, *FR* foramen rotundum, *VC* vidian canal, *CC* carotid canal

### Age distribution

Mean age of the study sample was 40.78 ± 14.44 (41.03 ± 14.95 in females and 40.53 ± 13.96 in males) years. The study group was divided into age groups in order to emphasise the age-related change more clearly and in detail, and also to make it visually more understandable. Three groups were created according to ages respectively as Group I (18–32 years) with 196 (32.7%) patients, Group II (33–47 years) with 206 patients and Group III (48–82 years) with 198 (33%) patients. The frequencies of the SSSP types, right and left CSSP types, the protrusion and dehiscence of adjacent structures for each age group were shown in Table 6. The number of presellar and sellar types of the SSSP were increased in Group III, whereas postsellar type was markedly decreased with increasing age (*p* = 0.033). We found that the frequency of the CSSP types with combined pneumatization (Type IV and V) decreased with increasing age. Also, there was a significant difference between these age groups according to both right and left sides (*p* = 0.029, *p* = 0.044) (Table 6).

**Table 6:** Distribution of the study sample variants according to the age groups

		Group I (18–32 years)	Group I (33–47 years)	Group I (48–82 years)	*p*
Sagittal SSP	Conchal	0 (0%)	2 (0.7%)	0 (0%)	**0.033** *
	Presellar	4 (1.3%)	6 (2%)	10 (3.3%)	
	Sellar	27 (9%)	25 (8.3%)	38 (12.7%)	
	Postsellar	67 (22.3%)	71 (23.7%)	50 (16.7%)	
Right coronal SSP	Type 0	45 (15%)	55 (18.4%)	67 (22.3%)	**0.029** *
	Type I	12 (4%)	15 (5%)	7 (2.3%)	
	Type II	1 (0.3%)	0 (0%)	1 (0.3%)	
	Type III	4 (1.3%)	6 (2%)	1 (0.3%)	
	Type IV	19 (6.3%)	15 (5%)	7 (2.3%)	
	Type V	20 (6.7%)	15 (5%)	10 (3.3%)	
Left coronal SSP	Type 0	40 (13.6%)	50 (16.6%)	68 (22.6%)	**0.044** *
	Type I	10 (3.3%)	12 (4%)	4 (1.3%)	
	Type II	0 (0%)	1 (0.3%)	0 (0%)	
	Type III	4 (1.3%)	5 (1.7%)	1 (0.3%)	
	Type IV	23 (7.7%)	17 (5.7%)	12 (4%)	
	Type V	22 (7.3%)	18 (6%)	13 (4.3%)	
OC protrusion	Absent	45 (15%)	55 (18.3%)	60 (20%)	**0.021** *
	Present	57 (19%)	53 (17.7%)	30 (10%)	
OC dehiscence	Absent	55 (18.3%)	52 (17.3%)	56 (18.7%)	0.144
	Present	50 (16.7%)	54 (18%)	33 (11%)	
FR protrusion	Absent	52 (17.3%)	63 (21.1%)	53 (17.7%)	0.540
	Present	52 (17.3%)	40 (13.3%)	40 (13.3%)	
FR dehiscence	Absent	80 (26.7%)	75 (25%)	76 (5.3%)	0.225
	Present	23 (7.7%)	32 (10.7%)	14 (4.6%)	
VC protrusion	Absent	31 (10.3%)	45 (15%)	51 (17%)	**0.001** *
	Present	78 (26%)	60 (20%)	35 (11.7%)	
VC dehiscence	Absent	49 (16.3%)	56 (18.7%)	56 (18.7%)	0.233
	Present	50 (16.6%)	51 (17%)	38 (12.7%)	
CC protrusion	Absent	48 (16%)	49 (16.3%)	53 (17.7%)	**0.048** *
	Present	60 (20%)	56 (18.7%)	34 (11.3%)	
CC dehiscence	Absent	84 (28%)	82 (27.4%)	75 (25%)	0.237
	Present	13 (4.3%)	25 (8.3%)	21 (7%)	

*Statistically significant p value shows the results of ANOVA test, *SSP* sphenoid sinus pneumatization types, *OC* optic canal, *FR* foramen rotundum, *VC* vidian canal, *CC* carotid canal

The frequencies of protrusion and dehiscence were evaluated according to age groups, and it was observed that these phenomena decreased with increasing age. However, the differences in frequencies between age groups were not significant, with the exception of the frequencies of OC, VC, and CC protrusion, which were found to be statistically significant (*p* = 0.021, *p* = 0.001, *p* = 0.048) (Table 6). The results of the binary logistic regression analysis for an age that is the only continuous variable in this study, was shown in Table 7. In general, the probability of having postsellar type, Type IV and V CSSP, the protrusion and dehiscence of OC, FR, VC, and CC decreased with increasing age. Increased age was significantly associated with a lower probability of having postsellar type, Type IV and V CSSP, the protrusion of OC, VC, and CC (*p* = 0.033, *p* = 0.044, *p* = 0.009, *p* = 0.000, *p* = 0.047) (Table 7).

**Table 7:** Binary logistic regression analysis of an age risk factor for having adjacent variations

Dependent Variable	β	S.E.	Wald	*p*	Odds Ratio	95% CI
OC Protrusion	-0.022	0.009	6.851	**0.009** *	0.978	(0.962–0.994)
OC Dehiscence	-0.012	0.009	1.786	0.181	0.988	(0.971–1.006)
FR Protrusion	-0.012	0.010	1.567	0.211	0.988	(0.970–1.007)
FR Dehiscence	-0.001	0.013	0.006	0.937	0.999	(0.974–1.024)
VC Protrusion	-0.041	0.009	19.797	**0.000** *	0.960	(0.943–0.977)
VC Dehiscence	-0.014	0.009	2.273	0.132	0.986	(0.968–1.004)
CC Protrusion	-0.026	0.008	4.632	**0.047** *	0.984	(0.968-1.000)
CC Dehiscence	0.016	0.013	1.562	0.211	1.016	(0.991–1.042)

* Statistically significant p value, *OC* optic canal, *FR* foramen rotundum, *VC* vidian canal, *CC* carotid canal

Overall, the *k* coefficient of interobserver variability for all observations ranged between 0.75 and 1.0 (good agreement).

## Discussion

Having the largest pneumatization downwards or laterally to varying degrees among the paranasal sinuses and displaying anatomical proximity to adjacent neurovascular structures, the SS is of great interest to surgeons to avoid iatrogenic complications. Previous studies investigated the possible relationships of the SSP with the protrusion and dehiscence of adjacent bony structures [1, 3, 8, 13]. To the best of our knowledge, there is no study that has comprehensively taken into the consideration the possible relation among the CSSP and SSSP types, and their impact on the protrusion or dehiscence of the adjacent bony structures in a larger sample size. It is well known that the increased degree of SSP was significantly associated with the increased frequency of the OC, FR, VC, and CC protrusions, leading to these structures susceptible to iatrogenic injury. Studies focusing on this association are of great value to describe these variations preoperatively for a correct planning of surgical intervention and preventing iatrogenic complications [1–16].

The present study aimed to provide data concerning the prevalence of SSSP and CSSP types and the individualized prevalence of the protrusion and dehiscence of the adjacent structures based on SSSP and CSSP. It also examined the relationship of these factors with age, gender, and laterality. In this study, the most observed SSSP type was the postsellar type (62.7%), followed by the sellar type (30%) and the presellar type (6.6%) which is similar to results of some studies [5, 10, 11, 15, 19, 20]. Meanwhile, our results did not concur with the studies which reported the sellar type as the most prevalent pneumatization followed by the presellar type [1, 12–14, 18, 21]. Obviously, the results obtained regarding the rates of SSSP types vary considerably depending on the different classification patterns and ethnic variations.

In the context of the CSSP pattern, previous studies have employed the Vaezi classification [7], which delineates three distinct types: pre-vidian, pre-rotundum, and post-rotundum. Conversely, more recent studies have utilized the Wang classification [9]. The VR line, which connects the medial edge of the VC and the extracranial end of the FR, is used as the boundary between the SS and the lateral parts of the sphenoid bone, including the GWS, PP, and both (full lateral or combined). In Asal et al. [2], Badran et al. [19], and Şimşek et al. [20]’s studies the incidences of CSSP types were reported as 21.8%, 39.4%, and 45.8% pre-vidian, 37.5%, 33.2%, and 22.4% pre-rotundum, 40.7%, 27.3%, and 31.8% post-rotundum, respectively. According to Wang classification, Wang et al. [9] and Bilgir et al. [10] reported the prevalences as 5.5% and 43.3% lateral, 10.5% and 6.7% ACP, 5% and 26.4% PP, 35.5% and 23.6% combined type CSSP. In Azab et al. [22] study, the CSSP types were reported as ACP pneumatization (2.1%), PP pneumatization (2.1%), GWS pneumatization (4.7%), full lateral pneumatization (11.3%), and combined pneumatization (56.9%).

On the other hand, Refaat et al. [1] and Hewaidi et al. [8] defined three planes; one horizontal plane crossing the ACP, one horizontal plane crossing the PP, and a vertical plane crossing the FR similar to our study. Based on this classification, Refaat et al. [1], Fatihoglu et al. [3], Aijaz et al. [5], Hewaidi et al. [8], Degaga et al. [13], and Sagar et al. [21] reported three types of the CSSP, in which the incidence of PP pneumatization was found as 29%, 26.5%, 5.7%, 23.6%, 15%, and 44%, ACP pneumatization as 15.3%, 27.6%, 23.6%, 26.3%, 18%, and 31.33%, GWS pneumatization as 20%, 22.9%, 28.6%, 12.3%, 16.5%, and 16.67%, respectively. They did not describe any combined or full lateral type pneumatization. So, our results were not comparable with the findings of these studies. In the course of our study, five distinct types of the CSSP were identified in 214 patients (71.3%). The most prevalent types were Type IV and Type V, which were observed in 23.6% and 21.7% of cases, respectively. These types were characterised by the presence of multiple extensions within the same side sinus. In contrast, Type II was the least common type, accounting for only 1% of cases. This type was characterised by a sinus extension that terminated below a horizontal line passing through the VC. In these cases, Type I, II, and III CSSP were observed to occur more frequently on the right side in 32, 2, and 7 patients, respectively. Type IV was observed to occur more frequently on the left side in 30 patients. Type V was observed to occur more frequently bilaterally in 33 patients. In females, Type IV was observed to occur more frequently on the right side, while Type I and Type IV were observed to occur more frequently on the left side than in males. In males, Type V CSSP was more prevalent on both the right and left sides than in females. It is evident that the results obtained regarding the rates of the SSSP and CSSP types exhibit considerable variability. The disparity in the rates observed in these studies may be attributed to differences in classification patterns, variations in radiological techniques or study design, and also the ethnic and anatomical variations. Furthermore, the inter-observer variability in the study groups may result in difficulties in comparison.

In this current study, the protrusion of the OC, FR, VC, and CC were observed in 46.7%, 44%, 57.7%, and 50% of subjects, whereas the prevalence of the OC, FR, VC, and CC dehiscence were 45.6%, 23%, 46.3%, and 19.7%, respectively. Unilateral/bilateral OC and CC protrusion were detected more in males (56.6% and 58.7%) compared to females (36.6% and 41.3%) (for OC; *p* = 0.013, χ2 = 6.178, and for CC; *p* = 0.005, χ2 = 7.792). In addition, unilateral and bilateral VC dehiscence were detected more in females (54%) compared to males (38.7%) (*p* = 0.041, χ2 = 4.164). As shown in Table 8, the reported prevalence of protrusion and dehiscence of adjacent bony structures varies widely according to the ethnicity of the study groups and the anatomical identification or classification system used.

**Table 8:** Comparison of the prevalence of protrusion and dehiscence of adjacent structures with other studies

Studies	OCP	OCD	FRP	FRD	VCP	VCD	CCP	CCD
Hewaidi 2008 (300 CT)	35.6%	30.6%	24.3%	13%	27%	37%	41%	30%
Refaat 2020 (170 CT)	50.6%	4.1%	21.1%	2.4%	30%	2.9%	47.6%	4.1%
Degaga 2020 (200 CT)	9.5%	15.5%	12.5%	12.5%			18.5%	12%
Fatihoğlu 2021 (1003 CT)	18.6%	18.6%	25%	11%	26.7%	6.7%	31.9%	21.9%
**Our study 2024 (300CT)**	46.7%	45.6%	44%	23%	57.7%	46.3%	50%	19.7%
Asal 2018 (300 CT)	74%	10.7%					58.2%	27.7%
Raseman 2020 (81 CT)	17%	6%					30%	5%
Thakur 2020 (400 CT)				6.4%		40.5%	12.9%	
Movahhedian 2021 (500CT)	40.4%	64.5%					37%	42.8%
Fadda 2022 (230CT)	13%	1.5%					26.3%	0.4%
Aijaz 2023 (300CT)	38%	16%					49%	9.3%
Serindere 2023 (150CT)	28.7%	18.7%					40.7%	40.7%
Tavakoli 2023 (97 CBCT)	44.3%						34%	
Şimşek 2024 (262 CT)	39.4%				53%		24.4%	

*OCP* and *OCD* optic canal protrusion and dehiscence, *FRP * and *FRD* foramen rotundum protrusion and dehiscence, *VCP* and *VCD* vidian canal protrusion and dehiscence, *CCP* and *CCD* carotid canal protrusion and dehiscence

Most of previous studies emphasized that there is a strong positive correlation between the degree of the SSP and the overall prevalence of the protrusion and dehiscence of adjacent neurovascular structures [1, 3, 8, 13]. With regard to the individualized prevalence of the protrusion and dehiscence of these structures in accordance with SSSP and CSSP patterns, our findings indicate that the degree of aeration in the SSSP and CSSP types is interdependent. Additionally, the rates of protrusion and dehiscence of adjacent bony structures are primarily influenced by the degree of SSP. Our findings indicate that the protrusion of all adjacent structures into the sinus cavity is most commonly observed in conjunction with postsellar type and Type V CSSP (*p* < 0.005). Conversely, the dehiscence of the FR and CC does not appear to be strongly associated with the increased pneumatization of the SS (*p* > 0.05). Furthermore, we observed that bilateral protrusion of the OC, VC, and CC was more prevalent in the postsellar type (81.4%, 85.2%, 96.5%), while unilateral protrusion of the FR was more common in the postsellar type (91.6%). In contrast to the aforementioned protrusion rates, the prevalence of unilateral (OC 61%, FR 56.8%, VC 64.5%, CC 55%) and bilateral dehiscence (OC 78.4%, FR 55.6%, VC 84.4%, CC 57.9%) of all adjacent structures was found to be significantly higher in the postsellar type. These findings are in line with previous studies conducted by Refaat et al. [1], Fatihoglu et al. [3], and Aijaz et al. [5], but not compared with results of other studies because of the lack of the individualized prevalence of the protrusion and dehiscence of the adjacent structures based on SSSP.

A review of the literature reveals a paucity of studies that have examined the relationship between the protrusion and dehiscence of adjacent structures and CSSP types. In this current study, among the 140 patients with OC protrusion, 105 (75%) exhibited Type V, 14 (10%) exhibited Type IV, 9 (6.4%) exhibited Type III, and 12 (8.6%) exhibited Type I, respectively. Notably, no patient exhibited Type II CSSP. As illustrated in Fig. 6, analogous outcomes were observed with respect to the FR, VC, and CC protrusion rates. Conversely, the frequencies of OC, FR, and CC dehiscence were found to be almost equal in Type IV and V, whereas VC dehiscence was observed in 139 (46.3%) patients, of whom 75 (54%) were coexisted with Type V, 48 (34.5%) with Type IV, 6 (4.3%) with Type III, 1 (0.7%) with Type II, and 9 (6.5%) with Type I, respectively. A comprehensive analysis of the association between the rates of adjacent structures variations and CSSP types could not be found in the existing literature. Our results may therefore provide a valuable contribution to this field of study. The findings of this study indicate that well-pneumatized SS, in particular postsellar type, Type IV and V CSSP, are associated with the highest likelihood of protrusion and dehiscence of adjacent structures. This suggests that an increase in the degree of SSP is associated with an increased likelihood of OC, FR, VC, and CC visibly traversing the sinus, thereby rendering them more susceptible to injury. In the event of a considerable degree of pneumatization, it becomes necessary to ascertain the size of the surgical window and the probability of iatrogenic injury to adjacent structures prior to FESS. In the absence or minimal pneumatization of SS, the knowledge of the dimensions of substantial bone drilling may prove beneficial in accessing the skull base and in reducing the preoperative risk.

Nevertheless, previous studies have only documented the lower incidence of aberrant SSP, namely the protrusion and dehiscence of adjacent structures in later ages. This observation was not statistically significant, and no study has yet analysed the influence of ageing in detail [17, 23, 24]. Our findings indicate that the SSP declines gradually with age. Furthermore, a significant negative correlation was observed between ageing and the incidence of protrusion of the OC, VC, and CC. Increased age was significantly associated with a lower probability of having postsellar type, Type IV and V CSSP, the protrusion of OC, VC, and CC. This outcome may help surgeon to understand changes in both SS and surrounding structures with aging. This is linked with bone resorption, deterioration and not of expansion, and also with atrophic changes in bone depending on vessel hypoperfusion [17, 24].

The current study has certain limitations. First, our results comprise the data belonging to healthy Turkish individuals. Due to lack of groups including healthy controls and patients with pathological conditions and postoperative outcomes, our sample may not be representative of the whole population. Second, this retrospective study was conducted in a single center with adult patients, it can not be possible to make a consequence for different populations with a higher sample size. Even though our study sample size is huge compared to other studies, multi-center studies with patients from different racial groups might be a lot better to demonstrate the impact of ethnicity and and comorbidities, so the results may be representative of the entire population.

## Conclusion

Unlike previous studies that solely examined the overall prevalence of protrusion and dehiscence of adjacent structures with regard to the SSP, the findings of the present study demonstrated that the individualized prevalence of protrusion and dehiscence in OC, FR, VC, and CC exhibited an upward trajectory in conjunction with augmented pneumatization, in particular postsellar type and Type IV and V CSSP. Our results indicated that well-pneumatized SS associated with the highest likelihood of protrusion and dehiscence of OC, FR, VC, and CC. Furthermore, no significant correlation was observed between these qualitative variables and gender, with the exception of the VC dehiscence, the protrusion of OC and CC. No notable differences were identified with respect to laterality. Our study also demonstrated that the probability of having postsellar type, Type IV and V CSSP, as well as the protrusion of OC, VC, and CC, decreased with increasing age. Different classification patterns, differences in radiological techniques or study design, as well as ethnic and anatomical variations may account for the related/unrelated causes of findings in this study with previous studies. Our findings are expected to serve as fundamental data to increase the safety of transsphenoidal procedures. In order to achieve successful therapeutic effects through a safe and accurate surgical procedure with a minimal risk of iatrogenic complications, it is essential for the surgeon to be aware of the significant association between the SSP and variations of adjacent structures, as well as the impact of age.

## Data Availability

No datasets were generated or analysed during the current study.
